# The US Affordable Care Act: Reflections and directions at the close of a decade

**DOI:** 10.1371/journal.pmed.1002752

**Published:** 2019-02-26

**Authors:** Adrianna McIntyre, Zirui Song

**Affiliations:** 1 PhD Program in Health Policy, Harvard University, Cambridge, Massachusetts, United States of America; 2 Department of Health Care Policy, Harvard Medical School, Boston, Massachusetts, United States of America; 3 Department of Medicine, Massachusetts General Hospital, Boston, Massachusetts, United States of America

## Abstract

In this month's Editorial, PLOS Medicine Academic Editor Zirui Song and his colleague Adrianna McIntyre discuss outcomes and possible futures for the United States Affordable Care Act as it nears the ten year mark.

Nearly nine years after its passage, the Affordable Care Act (ACA) remains at the forefront of public policy debate. The law is persistently contentious as a matter of public opinion, but represents a historic achievement in United States healthcare reform. While it was incremental in many respects—health insurance plans for the vast majority of Americans were relatively unchanged—the ACA left an indelible mark on the healthcare system through its expansion of insurance coverage and efforts to improve the healthcare delivery system. In the past decade, the country has witnessed a substantial decline in the number of uninsured individuals, while other elements of the law have sought to make inroads into affecting the cost and quality of care [1]. Yet looking forward, the ACA continues to face challenges that make its abiding impact and legacy uncertain.

## What the ACA did for insurance expansion

The ACA expanded insurance coverage in two principal ways. First, it created health insurance marketplaces at the state level on the premise of competition and choice; individuals could compare similar coverage options and choose among competing plans. The health law also provided low-income individuals and households up to 400% of the federal poverty line with subsidies to help them purchase insurance. Second, the ACA expanded eligibility for the Medicaid program to individuals and families with incomes up to 138% of the federal poverty line—about US$35,000 for a family of four. Since the law’s implementation in 2010, the number of uninsured people in the country has fallen by about 20 million [2].

The ACA reshaped private insurance in other important ways. It established new minimum federal consumer protections; of note, insurers were prohibited from discriminating on the basis of health status—they could not turn people away or charge higher premiums due to pre-existing medical conditions. A set of 10 “essential health benefits” was defined. Annual and lifetime limits on covered health benefits were abolished. The law’s dependent coverage provision enabled children up to age 26 to stay on their parents’ insurance, benefiting between 2 and 3 million young people [3].

Expansions in health insurance were aided by complementary policies that encouraged people to enroll in coverage. Federal tax credits that reduced the financial burden of monthly premiums—and, in some cases, reduced cost-sharing—made plans on the marketplaces more appealing to low-income consumers. The subsidies functioned as a carrot that was balanced by a stick: the ACA’s individual mandate required people to get covered or else pay a tax penalty. However, the Tax Cuts and Jobs Act effectively eliminated this policy by lowering the penalty for not having health insurance to US$0 beginning in 2019.

When it launched in 2014, this type of regulated individual market was new terrain for most insurers. They were responsible for projecting the likely healthcare costs of people who would elect to take up coverage, with limited experience to guide these estimates. To assuage insurers’ concerns about enrolling unexpectedly sick (and expensive) populations, the ACA implemented federal protections through three programs: risk corridors, reinsurance, and risk adjustment. The first two were temporary; they expired after three years but gave insurers an opportunity to find their footing and price their products accurately. Risk adjustment is a permanent program, intended to mitigate against insurers selecting healthier enrollees and avoiding sicker populations.

## Challenges to the ACA

The law has endured numerous legislative challenges following its passage. The House of Representatives advanced over 50 bills to repeal the ACA in whole or in part, with the Senate voting on a subset of them [4]. These started out as largely symbolic—a presidential veto was virtually guaranteed while President Obama was in office—but began to pose an existential threat to the ACA under a unified Republican government that held power during the first two years of the Trump administration. The narrow 49-to-51 vote defeat of the last prominent repeal effort in the summer of 2017 illustrated the tenuous grounds upon which the law sat in the previous Congress. However, its survival was also a testament to its legislative durability; the political challenge of withdrawing health benefits shared across different constituencies has thus far been insurmountable, despite lukewarm public opinion on the law.

Proponents of the ACA have identified some regulatory actions by the Trump administration as unilateral efforts to undermine the law. For example, terminating funding for cost-sharing reductions, which are supplemental subsidies available to some low-income enrollees, led to fears about destabilizing the markets and increasing the ranks of the uninsured. Cutting resources allocated to enrollment outreach and education have raised similar concerns. Recent changes to insurance regulations will likely make plans that bypass the ACA’s consumer protections more common. Moreover, the administration has made it easier for states to modify their Medicaid programs in ways that could lower enrollment (by requiring nondisabled beneficiaries to work in order to qualify for benefits, for example). Its proponents have championed these changes as efforts to promote consumer choice and state innovation.

Other serious threats to the law’s sustainability have come from the courts. A landmark 2012 Supreme Court decision scaled back the Medicaid expansion from a nationwide mandate to a state option. To date, 14 states have declined to expand their Medicaid programs (although this number has gradually decreased in recent years). Another challenge sought to roll back subsidies on the ACA marketplaces. Still other litigation concerning regulations related to contraceptive coverage is ongoing. Perhaps the ACA’s greatest lingering existential threat comes from a late-2018 district court ruling in Texas. The judge in this case ruled that the zeroed-out mandate is unconstitutional—and, moreover, that the mandate is not severable from the rest of the ACA, meaning that the rest of the law would need to fall with it. The case is now within the appeals process and could end up before the Supreme Court.

## Looking forward: 2019 and beyond

The prospects for near-term repeal have diminished with Democrats taking control of the House of Representatives, but the ACA has not receded from the public debate. On the contrary, healthcare ranked among voters’ most important issues in the 2018 midterms. The administration and new Congress will need to decide whether to leave the law alone or modify it. Additionally, attempts to weaken the law through regulatory channels will be subject to increased scrutiny now that Democrats have more congressional oversight.

Opportunities for bipartisan legislation to stabilize the law appear slim. The leading Republican and Democrat of the Senate Health, Education, Labor, and Pensions Committee coauthored a modest marketplace stabilization bill in 2017 that would have provided funding for cost-sharing reductions, increased funding for enrollment outreach and assistance, and made other minor tweaks to the law. However, Democrats may be reticent to revive that bill, as insurers in many states addressed the cost-sharing reductions issue in a way that made insurance more affordable for subsidized enrollees. A more ambitious stabilization bill was introduced by House Democrats in 2018, which would increase the availability of marketplace subsidies by lifting the income cap (currently at 400% of poverty), reverse certain regulations by the current administration, and provide more funding for consumer outreach and assistance. The prospects of this bill are dim without support from across the political aisle.

Perhaps more fundamental for the future direction of health policy, public opinion on the role of government in healthcare is evolving. In 2013, 42% of Americans believed that it is the responsibility of the government to ensure that all Americans have coverage; that number rose to 60% in 2017 [5]. Support for “Medicare for All” proposals has also climbed in recent surveys, though these opinions have been malleable to follow-up questions. These public opinion trends suggest that a growing share of Americans may be receptive to proposals that move the ACA in a more progressive direction.
